# Comparison of DNA testing strategies in monitoring human papillomavirus infection prevalence through simulation

**DOI:** 10.1186/s12879-016-1969-1

**Published:** 2016-11-07

**Authors:** Carol Y. Lin, Ling Li

**Affiliations:** 1 Centers for Disease Control and Prevention, Atlanta, GA USA; 2 United Arab Emirates University, Al Ain, UAE

**Keywords:** HPV prevalence, PCR, HPV clinical test, HPV vaccine effectiveness, Cervical cancer screening

## Abstract

**Background:**

HPV DNA diagnostic tests for epidemiology monitoring (research purpose) or cervical cancer screening (clinical purpose) have often been considered separately. Women with positive Linear Array (LA) polymerase chain reaction (PCR) research test results typically are neither informed nor referred for colposcopy. Recently, a sequential testing by using Hybrid Capture 2 (HC2) HPV clinical test as a triage before genotype by LA has been adopted for monitoring HPV infections. Also, HC2 has been reported as a more feasible screening approach for cervical cancer in low-resource countries. Thus, knowing the performance of testing strategies incorporating HPV clinical test (i.e., HC2-only or using HC2 as a triage before genotype by LA) compared with LA-only testing in measuring HPV prevalence will be informative for public health practice.

**Method:**

We conducted a Monte Carlo simulation study. Data were generated using mathematical algorithms. We designated the reported HPV infection prevalence in the U.S. and Latin America as the “true” underlying type-specific HPV prevalence. Analytical sensitivity of HC2 for detecting 14 high-risk (oncogenic) types was considered to be less than LA. Estimated-to-true prevalence ratios and percentage reductions were calculated.

**Results:**

When the “true” HPV prevalence was designated as the reported prevalence in the U.S., with LA genotyping sensitivity and specificity of (0.95, 0.95), estimated-to-true prevalence ratios of 14 high-risk types were 2.132, 1.056, 0.958 for LA-only, HC2-only, and sequential testing, respectively. Estimated-to-true prevalence ratios of two vaccine-associated high-risk types were 2.359 and 1.063 for LA-only and sequential testing, respectively. When designated type-specific prevalence of HPV16 and 18 were reduced by 50 %, using either LA-only or sequential testing, prevalence estimates were reduced by 18 %.

**Conclusion:**

Estimated-to-true HPV infection prevalence ratios using LA-only testing strategy are generally higher than using HC2-only or using HC2 as a triage before genotype by LA. HPV clinical testing can be incorporated to monitor HPV prevalence or vaccine effectiveness. Caution is needed when comparing apparent prevalence from different testing strategies.

## Background

Cervical cancer is the third most common cancer among women and the second most frequent cause of cancer-related deaths, accounting for approximately 300,000 deaths annually worldwide [1]. More than 80 % of these cervical cancer related deaths occurs in low-resource countries [2]. Human papillomavirus (HPV) infections have been identified as a necessary cause of cervical cancer [3–5]. Screening and vaccination are the best strategies for reducing cervical cancer incidence. HPV-clinical DNA testing has been considered as a more cost- effective and feasible approach [3]. Since HPV vaccination was introduced in 2006, many developed countries also have initiated HPV immunization programs for adolescents [6–8]. While the high cost of HPV vaccination remains a barrier, HPV vaccine has been introduced in some developing countries successfully [9]. Because cervical cancer outcomes take years to observe, monitoring HPV infections has served as an early indication of vaccine effectiveness [7, 10–13]. In this study, we compare three HPV DNA testing strategies for monitoring HPV infection prevalence.

Molecular testing methods are available for detecting HPV infections for research or for clinical purposes. Polymerase chain reaction (PCR)-based DNA genotyping tests using target amplification technique can detect the existence of minute amounts of virus and have been considered as the “gold standard” for detecting infectious organisms [14, 15]. Linear Array (LA) genotyping assay is the commercialized version of PCR genotyping testing designed to standardize the PCR process for detecting existence of HPV DNA for research purposes. The LA genotyping assay is based on PCR amplification of a 450-bp sequence of the L1 region by using PGMY09/11 primer, hybridization of the amplified product to oligonucleotide probes, and their detection by colorimetric reaction [16]. LA can detect 37 HPV genotypes simultaneously. The test has been used in epidemiological and clinical research studies to detect HPV infections and is also the most widely used assay by HPV LabNet laboratories to monitor HPV infection prevalence for HPV vaccine effectiveness [17]. By comparison, commercially available Digene Hybrid Capture 2 (HC2) clinical-HPV assay uses the signal amplified hybridization microplate-based technique to detect HPV DNA presence. The HC2 clinical HPV test is an aggregate test that detects 13 high-risk (oncogenic) types (HPV16, 18, 31, 33, 35, 39, 45, 51, 52, 56, 58, 59, and 68), and because of cross-reactivity, it can also detect HPV66. HC2 is designed to detect high enough viral loads (i.e., clinically relevant viral loads), and it has been used as a co-test with the Papanicolaou smear test or a clinical test alone for screening women likely to develop or who have already developed cervical cancer. The HC2 assay has also been used as a reference test in studies evaluating newly developed clinical-HPV assays [18, 19].

PCR genotyping test has been used to monitor HPV infection prevalence and vaccine effectiveness for research purposes because of its high analytical sensitivity. Women with positive PCR genotyping test results typically are neither informed nor referred for colposcopy, and the PCR processing procedures can be complex and labor-intensive. Recently, a sequential testing strategy using HC2 as a triage and genotype only those HC2 positive specimens by LA PCR has been used to monitor HPV infections in China and England [20, 21]. Using PCR genotyping assays to monitor HPV infections is also not feasible in low-resource countries [22–24]. Alternatively, HC2 has been reported as a more feasible screening approach in low-resource countries [3, 23] because HC2 (particularly, *care*HPV, the developed countries version of HC2) does not require special facilities and is less labor-intensive than PCR-based methods [22, 25, 26]. Thus, knowing the performance of testing strategies incorporating HPV-clinical HC2, compared with using LA-only PCR testing, in measuring HPV infection prevalence will be informative for public health practice.

Since sensitivities and specificities of these testing strategies may not be easily obtained and the true HPV infection prevalence is normally not known, we use Monte Carlo simulation approach to compare different testing strategies. Simulation methods have been used for answering “what-if” questions. For example, IF the “true” prevalence level is “a” and IF the sensitivity/specificity of the test is “b”/”c”, the simulation will provide information on what we expect to measure as the apparent prevalence (estimate) and thus the difference (bias) between the “true” and apparent prevalence (estimate). In this study, we use the reported HPV prevalence from the US and Latin America as the “true” HPV prevalence level. What we try to answer is, if the “true” prevalence is at the level as the reported prevalence in the US and Latin America and we use the test with sensitivity and specificity as specified, what is the apparent prevalence based on the testing strategy (i.e., LA-only, HC2-only, and sequential testing by using HC2 as a triage and genotyped by LA for HC2-positive specimens)? What is the ratio of the apparent prevalence to the “true” prevalence?

## Methods

### Simulation setup

The Monte Carlo simulation approach similar to Lin et al. [27, 28] was extended to incorporate various testing strategies. Analytical sensitivity is the probability of truly infected subjects with a positive test result; analytical specificity is the probability of truly uninfected subjects with a negative result. Mathematical algorithms with designated values of “true” underlying type-specific prevalence, genotyping assay analytical sensitivity and specificity, and correlations among HPV types were used to generate data.” True” type-specific infectious statuses were determined on the basis of the designated type-specific prevalence. The genotyping test results were determined based on the basis of type-specific infection status and genotyping test performance (i.e., analytical sensitivity and specificity). Without loss of generalizability, the total number of subjects in each data set was set at 4,000, and correlations between HPV types were set at 0.06. For each simulated data set, there are “true” type-specific infectious status of 14 high-risk (oncogenic) types, the LA genotyping test results of 14 high-risk types and the HC2 result for each of the 4000 subject. Five hundred data sets were generated for each scenario. Mean and standard deviation of the “true” and apparent prevalence (estimates) of 500 data sets were calculated. Mathematical algorithms related to our simulation setup were given in the literature [27, 28].

**Fig. 1:**
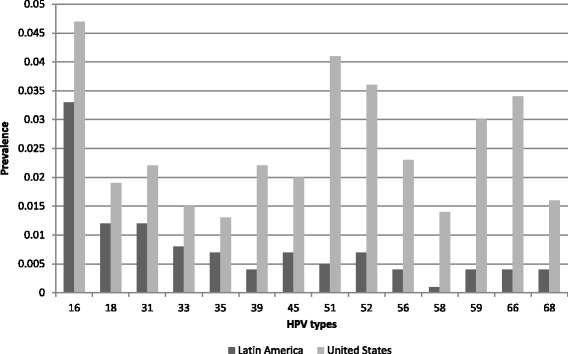
The reported type-specific prevalence of the 14 high-risk (oncogenic) types of the United States and Latin America

To simulate scenarios on the basis of both high- and low-resource regions, the reported prevalence of the 14 high-risk HPV types for the United States and Latin America were designated as the known “true” underlying type-specific prevalence (Fig. 1) [29, 30]. HPV type-specific prevalence in Latin America was lower than in the United States, and the relative HPV infection prevalence was also different.

LA genotyping assay (type-specific) analytical sensitivity and specificity were initially set as (0.95, 0.95) to reflect the scenarios where specimens were carefully handled and PCR procedures were conducted adequately. Since various factors (i.e., precisely executing processing procedures, number and quality of virus genomes presented in the samples, etc.) could affect the performance of LA, LA genotyping assay analytical sensitivity and specificity were subsequently varied from (0.95, 0.95) to (0.95, 0.90), (0.95, 0.85), (0.90, 0.90), (0.90, 0.85), (0.85, 0.85), (0.80, 0.80). The idealistic scenario, LA genotyping assay analytical performance reached (1.00, 1.00), was also considered.

To obtain HC2 results, we based our study on the test results of 8,403 women participating in the U.S. NHANES (2003–2010) study with both LA and HC2 test conducted [9]. Forty-five percent of subjects with at least one LA-positive test among 14 high-risk HPV types also tested positive by HC2, and 93 % of subjects with all LA type-specific negative results also tested negative by HC2. This was equivalent to that the analytical sensitivity of HC2 in detecting any of the 14 high-risk HPV types was approximately 50 % lower than LA, but the analytical specificity was similar.

For each simulated data set, the data included the “true” type-specific infectious status of 14 high-risk types, the LA genotyping test results of 14 high-risk types and the HC2 result of the 4,000 subject. Using the simulated data, the “true” prevalence, estimated prevalence and estimated-to-“true” prevalence ratios from various testing strategies based on the following definition were calculated.

### “True” and estimated infection prevalence

Four outcome measures: 14 oncogenic high-risk types, 2 vaccine-associated high-risk types (HPV16 or HPV18 based on bivalent and quadrivalent HPV vaccine), type-specific HPV16, and type-specific HPV 18 were considered. HPV 16 and HPV 18 infections account for approximately 70 % of invasive cervical cancer globally [31, 32]. The “true” prevalence of each outcome measure was defined as the proportion of subjects having HPV infections. The “true” infection status of the four outcome measures were defined as follows: for each subject, the “true” positive infection status of the 14 high-risk types or the 2 vaccine-associated high-risk types was defined as having at least one of the 14 high-risk or the 2 vaccine-associated high-risk types, respectively. The “true” positive type-specific infection status of HPV16/HPV18 was defined as having type-specific infection of HPV16/HPV18.

Apparent prevalence (estimates) of each outcome measure was defined as a proportion of subjects with positive test results from different testing strategies. For the LA-only testing strategy, specimens taken from all subjects were genotyped by LA, therefore, the type-specific results of 14 HPV type were available. A positive LA test outcome of the 14 high-risk types and the 2 vaccine-associated high-risk types was defined as having at least one positive LA type-specific result of the 14 high-risk types and two positive vaccine-associated high-risk types, respectively. A positive test outcome of individual type-specific HPV16 or HPV18 was defined as having a positive HPV16 or HPV18 LA genotyping result, respectively.

For the HC2 clinical HPV testing-only strategy, specimens of all subjects were tested by HC2, therefore, HC2 results were available for all subjects. A positive HC2 test result was defined as subjects with at least one of the 14 high-risk HPV types. Since HC2 test result was an aggregate result, no type-specific result was available.

For the sequential testing strategy, specimens of all subjects were tested by HC2 first, and only those specimens with positive HC2 results were genotyped by LA. Therefore, for HC2 negative subjects, the LA type-specific results were not available. A positive sequential test outcome of the 14 high-risk types was defined as having both HC2-positive and at least one positive LA type-specific result of the 14 high-risk types. Similarly, a positive sequential test outcome of the two vaccine-associated high-risk types was defined as having a positive HC2 result and at least one positive LA type-specific result of two vaccine-associated high-risk types. A positive sequential test outcome of HPV16/HPV18 was defined as having positive HC2 and HPV16/HPV18 type-specific LA results.

The estimated-to-true prevalence ratio was calculated to examine how well prevalence estimates that were based on different testing strategies measured the designated “true” prevalence. A ratio >1 indicated the prevalence estimate overestimated the designated “true” prevalence. A ratio <1 indicated the prevalence estimate underestimated the designated “true” prevalence. To examine vaccine effectiveness, for demonstration purpose, the designated “true” type-specific prevalence of vaccine-associated high-risk types (i.e., HPV16 and HPV18) were reduced by 50 %. Percentage reductions of prevalence estimates of the 14 high-risk types, two vaccine-associated high-risk types, HPV16, and HPV18 that were based on different testing strategies were calculated.

## Results

Apparent prevalence (estimates) of the 14 high-risk HPV types by various testing strategies are given in Table 1. In the idealistic scenario, when the analytic sensitivity and specificity of LA genotyping assay reach (1.00, 1.00), as expected, the LA-only testing strategy performs the best. When LA genotyping assay sensitivity and specificity are ≤0.95 and ≤0.95, respectively, prevalence estimates from all three testing strategies generally overestimat designated infection prevalence of the 14 high-risk types. The prevalence estimate from the LA-only testing strategy is higher than the HC2-only or sequential testing strategies.

**Table 1:** Designated and estimated prevalence of the 14 high-risk virus types by Linear Array (LA)-only, Hybrid Capture (HC2)-only, and sequential testing strategies in the United States and Latin America

Genotyping assay performance	Time	Designated composite prevalence (SD)	LA-only		HC2-only		Sequential test	
			Composite prevalence estimate (SD)	Estimate-to-true ratio	Composite prevalence estimate (SD)	Estimate-to-true ratio	Composite prevalence estimate (SD)	Estimate-to-true ratio
United States								
Sen = 1.00 Spe = 1.00	Baseline	0.287 (0.007)	0.287 (0.007)	1.000	0.180 (0.006)	0.628	0.129 (0.005)	0.446
	Reduced	0.265 (0.007)	0.265 (0.007)	1.000	0.172 (0.006)	0.657	0.124 (0.005)	0.468
	% Reduction	7.6	7.6		0.3		3.9	
Sen = 0.95 Spe = 0.95	Baseline	0.287 (0.007)	0.612 (0.008)	2.132	0.303 (0.007)	1.056	0.275 (0.007)	0.958
	Reduced	0.265 (0.007)	0.602 (0.008)	2.272	0.299 (0.007)	1.128	0.271 (0.007)	1.023
	% Reduction	7.6	1.6		1.3		1.5	
Sen = 0.95 Spe = 0.90	Baseline	0.287 (0.007)	0.792 (0.006)	2.760	0.371 (0.008)	1.293	0.356 (0.008)	1.240
	Reduced	0.265 (0.007)	0.787 (0.007)	2.970	0.369 (0.008)	1.392	0.354 (0.008)	1.340
	% Reduction	8.0	0.6		0.5		0.6	
Sen = 0.90 Spe = 0.90	Baseline	0.287 (0.007)	0.788 (0.006)	2.746	0.370 (0.007)	1.289	0.355 (0.007)	1.237
	Reduced	0.265 (0.007)	0.783 (0.007)	2.955	0.368 (0.007)	1.389	0.352 (0.002)	1.328
	% Reduction	7.7	0.6		0.5		0.8	
Sen = 0.90 Spe = 0.85	Baseline	0.287 (0.007)	0.887 (0.005)	3.091	0.407 (0.008)	1.418	0.399 (0.008)	1.390
	Reduced	0.265 (0.007)	0.885 (0.005)	3.340	0.405 (0.008)	1.528	0.397 (0.008)	1.498
	% Reduction	8.0	2.5		0.5		0.3	
Sen = 0.85 Spe = 0.85	Baseline	0.287 (0.007)	0.885 (0.005)	2.979	0.407 (0.007)	1.418	0.399 (0.007)	1.390
	Reduced	0.265 (0.007)	0.883 (0.005)	3.332	0.405 (0.007)	1.528	0.397 (0.007)	1.498
	% Reduction	8.0	2.2		0.5		0.3	
Sen = 0.80 Spe = 0.80	Baseline	0.286 (0.007)	0.939 (0.004)	3.283	0.426 (0.008)	1.489	0.422 (0.008)	1.475
	Reduced	0.265 (0.007)	0.938 (0.004)	3.540	0.424 (0.008)	1.600	0.421 (0.008)	1.589
	% Reduction	8.0	1.2		4.7		0.2	
Latin America								
Sen = 1.00 Spe = 1.00	Baseline	0.104 (0.005)	0.104 (0.005)	1.000	0.110 (0.005)	1.058	0.048 (0.003)	0.461
	Reduced	0.084 (0.004)	0.084 (0.004)	1.000	0.103 (0.005)	1.220	0.039 (0.003)	0.464
	% Reduction	19.2	19.2		6.3		18.7	
Sen = 0.95 Spe = 0.95	Baseline	0.104 (0.005)	0.527 (0.008)	5.067	0.270 (0.007)	2.596	0.237 (0.007)	2.279
	Reduced	0.084 (0.004)	0.518 (0.008)	6.167	0.267 (0.007)	3.179	0.233 (0.006)	2.774
	% Reduction	19.2	1.7		1.1		1.7	
Sen = 0.95 Spe = 0.90	Baseline	0.104 (0.005)	0.749 (0.007)	7.202	0.355 (0.007)	3.413	0.338 (0.007)	3.250
	Reduced	0.084 (0.005)	0.745 (0.007)	8.869	0.353 (0.007)	4.202	0.335 (0.007)	3.988
	% Reduction	19.2	0.5		0.6		0.8	
Sen = 0.90 Spe = 0.90	Baseline	0.104 (0.004)	0.748 (0.007)	7.192	0.354 (0.008)	3.404	0.337 (0.008)	3.240
	Reduced	0.084 (0.004)	0.743 (0.007)	8.845	0.353 (0.008)	4.202	0.335 (0.008)	3.988
	% Reduction	19.2	0.7		0.3		0.6	
Sen = 0.90 Spe = 0.85	Baseline	0.104 (0.005)	0.867 (0.006)	8.337	0.400 (0.008)	3.846	0.390 (0.008)	3.750
	Reduced	0.084 (0.004)	0.865 (0.005)	10.298	0.399 (0.008)	4.750	0.390 (0.008)	4.643
	% Reduction	19.2	0.3		0.2		0	
Sen = 0.85 Spe = 0.85	Baseline	0.104 (0.005)	0.866 (0.005)	8.327	0.399 (0.007)	3.837	0.389 (0.007)	3.740
	Reduced	0.084 (0.004)	0.864 (0.005)	10.286	0.398 (0.007)	4.738	0.388 (0.007)	4.619
	% Reduction	19.2	0.2		0.2		0.2	
Sen = 0.80 Spe = 0.80	Baseline	0.104 (0.005)	0.930 (0.004)	8.942	0.424 (0.008)	4.077	0.419 (0.008)	4.029
	Reduced	0.084 (0.004)	0.929 (0.004)	11.060	0.423 (0.008)	5.036	0.418 (0.008)	4.976
	% Reduction	19.2	0.1		0.2		0.2	

Type-specific prevalence at baseline, in the United States: HPV16 = 0.047; HPV18 = 0.019; HPV31 = 0.022; HPV33 = 0.015; HPV35 = 0.013; HPV39 = 0.022; HPV45 = 0.020; HPV51 = 0.041; HPV52 = 0.036; HPV56 = 0.023; HPV58 = 0.014; HPV59 = 0.030; HPV66 = 0.034; and HPV68 = 0.016; in Latin America: HPV16 = 0.033; HPV18 = 0.012; HPV31 = 0.012; HPV33 = 0.008; HPV35 = 0.007; HPV39 = 0.004; HPV45 = 0.007; HPV51 = 0.005; HPV52 = 0.007; HPV56 = 0.004; HPV58 = 0.001; HPV59 = 0.004; HPV66 = 0.004; and HPV68 = 0.004

Reduced: HPV16 and HPV18 are reduced by 50 %

Abbreviations: *SD* standard deviation, *Sen* sensitivity, *Spe* specificity

Estimated-to-true prevalence ratios are larger in the Latin America scenarios than in the U.S. scenarios because the designated “true” type-specific infections are lower in Latin America. When LA genotyping assay sensitivity and specificity are equal to (0.95,0.95) and the designated individual type-specific infection prevalence rates of HPV16 and HPV18 are reduced by 50 %, the designated “true” composite infection prevalence of 14 high-risk types is decreased by 7.6 % in the United States and 19.2 % in Latin America. The estimated reductions of 14 high-risk types from the LA-only, HC2-only, and sequential testing strategies are similar (1.6 %, 1.3 %, and 1.5 % for the United States and 1.7 %, 1.1 %, and 1.7 % for Latin America) and much lower than the designated “true” percentage reduction.

When LA genotyping assay sensitivity and specificity are ≤0.95 and ≤0.95, respectively, using either the LA-only or the sequential testing strategy overestimates the designated “true” prevalence of the two vaccine-associated high-risk types (Table 2). The prevalence estimates using the LA-only testing strategy are higher than using the sequential testing strategy. Similarly, compared with the U.S. scenarios, estimated-to-true prevalence ratios are higher when the “true” prevalence is designated as the reported prevalence in Latin America. When the designated “true” type-specific prevalence of HPV16 and HPV18 are reduced by 50 %, composite prevalence estimates calculated on the basis of either the LA-only or the sequential testing strategy underestimate the designated reduction.

**Table 2:** Designated and estimated prevalence of two vaccine-associated high-risk types by Linear Array (LA)-only and sequential testing strategies in the United States and Latin America

Genotyping assay performance	Time	Designated composite prevalence (SD)	LA only		Sequential testing	
			Composite prevalence estimate (SD)	Estimate-to-true ratio	Composite prevalence estimate (SD)	Estimate-to-true ratio
United States						
Sen = 1.00 Spe = 1.00	Baseline	0.064 (0.004)	0.064 (0.004)	1.000	0.029 (0.003)	0.453
	Reduced	0.032 (0.003)	0.032 (0.003)	1.000	0.015 (0.002)	0.469
	% reduction	50	50		48	
Sen = 0.95 Spe = 0.95	Baseline	0.064 (0.004)	0.151 (0.006)	2.359	0.068 (0.004)	1.063
	Reduced	0.032 (0.003)	0.124 (0.005)	3.875	0.056 (0.004)	1.750
	% Reduction	50	17.9		17.6	
Sen = 0.95 Spe = 0.90	Baseline	0.064 (0.004)	0.287 (0.007)	4.484	0.107 (0.005)	1.672
	Reduced	0.032 (0.003)	0.213 (0.006)	6.656	0.096 (0.004)	3.000
	% Reduction	50	25.8		10.3	
Sen = 0.90 Spe = 0.90	Baseline	0.064 (0.004)	0.234 (0.007)	3.656	0.105 (0.005)	1.641
	Reduced	0.032 (0.003)	0.211 (0.006)	6.594	0.095 (0.005)	2.969
	% Reduction	0.50	9.8		9.5	
Sen = 0.90 Spe = 0.85	Baseline	0.064 (0.004)	0.314 (0.008)	4.906	0.142 (0.006)	2.219
	Reduced	0.032 (0.003)	0.294 (0.007)	9.188	0.132 (0.006)	4.125
	% Reduction	50	6.4		7.0	
Sen = 0.85 Spe = 0.85	Baseline	0.064 (0.004)	0.312 (0.007)	4.875	0.141 (0.005)	2.219
	Reduced	0.032 (0.003)	0.293 (0.008)	9.156	0.132 (0.005)	4.125
	% Reduction	50	42		7.0	
Sen = 0.80 Spe = 0.80	Baseline	0.064 (0.004)	0.385 (0.008)	6.016	0.173 (0.006)	2.703
	Reduced	0.032 (0.003)	0.371 (0.007)	11.594	0.167 (0.005)	5.219
	% Reduction	50	4.2		3.5	
Latin America						
Sen = 1.00 Spe = 1.00	Baseline	0.045 (0.003)	0.045 (0.003)	1.000	0.020 (0.002)	0.444
	Reduced	0.022 (0.002)	0.022 (0.002)	1.000	0.010 (0.002)	0.455
	% Reduction	51	5.1		5.0	
Sen = 0.95 Spe = 0.95	Baseline	0.045 (0.003)	0.135 (0.005)	3.000	0.060 (0.004)	1.333
	Reduced	0.022 (0.002)	0.116 (0.005)	5.273	0.052 (0.003)	2.364
	% Reduction	51	14.1		13.3	
Sen = 0.95 Spe = 0.90	Baseline	0.045 (0.003)	0.222 (0.007)	4.933	0.099 (0.005)	2.200
	Reduced	0.022 (0.002)	0.205 (0.006)	9.318	0.093 (0.005)	4.227
	% Reduction	51	7.6		6.1	
Sen = 0.90 Spe = 0.90	Baseline	0.045 (0.003)	0.219 (0.006)	4.867	0.099 (0.005)	2.200
	Reduced	0.022 (0.002)	0.204 (0.006)	8.870	0.092 (0.005)	4.000
	% Reduction	51	6.8		7.1	
Sen = 0.90 Spe = 0.85	Baseline	0.045 (0.003)	0.302 (0.007)	6.711	0.136 (0.005)	3.022
	Reduced	0.022 (0.002)	0.288 (0.007)	13.091	0.130 (0.005)	5.909
	% Reduction	51	4.6		4.4	
Sen = 0.85 Spe = 0.85	Baseline	0.045 (0.003)	0.300 (0.007)	6.667	0.135 (0.005)	3.000
	Reduced	0.022 (0.002)	0.288 (0.007)	13.091	0.129 (0.005)	5.864
	% Reduction	51	4.6		4.4	
Sen = 0.80 Spe = 0.80	Baseline	0.044 (0.003)	0.376 (0.007)	8.545	0.169 (0.006)	3.841
	Reduced	0.022 (0.002)	0.366 (0.007)	16.63	0.165 (0.006)	7.500
	% Reduction	50	2.7		2.4	

Type-specific prevalence at baseline, in the United States: HPV16 = 0.047 and HPV18 = 0.019; in Latin America: HPV16 = 0.033 and HPV18 = 0.012

Reduced: HPV16 and HPV18 are reduced by 50 %

Abbreviations: *SD* standard deviation, *Sen*, sensitivity, Spe, specificity

Designated “true” and estimated type-specific prevalence for HPV16 are displayed in Table 3 and for HPV18 in Table 4. When LA genotyping sensitivity and specificity reach (1.00 1.00), LA-only performs best. The sequential testing strategy typically underestimates the designated underlying prevalence. When LA genotyping assay sensitivity and specificity are ≤0.95 and ≤0.95, respectively, the prevalence estimates from the LA-only testing strategy generally overestimate the designated “true” infection prevalence of HPV16 or HPV18. For HPV16, in certain scenarios, the prevalence estimates from sequential testing strategies underestimate the designated prevalence. Similarly, reducing the designated individual type-specific prevalence of HPV16 and HPV18 by 50 % when using either the LA-only or the sequential testing strategy underestimates the designated reduction.

**Table 3:** Designated and estimated prevalence of HPV16 by Linear Array (LA)-only and sequential testing strategies in the United States and Latin America

Genotyping assay performance	Time	Designated composite prevalence (SD)	LA only		Sequential testing	
			Composite prevalence estimate (SD)	Estimate-to-true ratio	Composite prevalence estimate (SD)	Estimate-to-true ratio
United States						
Sen = 1.00 Spe = 1.00	Baseline	0.047 (0.003)	0.047 (0.003)	1.000	0.009 (0.002)	0.191
	Reduced	0.023 (0.002)	0.023 (0.002)	1.000	0.004 (0.001)	0.173
	% Reduction	51.1	51.1		50	
Sen = 0.95 Spe = 0.95	Baseline	0.047 (0.003)	0.092 (0.004)	1.958	0.028 (0.003)	0.596
	Reduced	0.023 (0.002)	0.071 (0.004)	3.087	0.021 (0.002)	0.913
	% Reduction	51.1	22.8		0.7	
Sen = 0.95 Spe = 0.90	Baseline	0.047 (0.003)	0.140 (0.006)	2.979	0.052 (0.004)	1.106
	Reduced	0.023 (0.002)	0.120 (0.005)	5.217	0.044 (0.003)	1.913
	% Reduction	51.1	14.3		15.4	
Sen = 0.90 Spe = 0.90	Baseline	0.047 (0.003)	0.138 (0.005)	2.936	0.051 (0.003)	1.085
	Reduced	0.023 (0.002)	0.119 (0.005)	5.174	0.044 (0.003)	1.913
	% Reduction	51.1	13.8		13.7	
Sen = 0.90 Spe = 0.85	Baseline	0.047 (0.003)	0.185 (0.006)	3.936	0.075 (0.004)	1.596
	Reduced	0.023 (0.002)	0.167 (0.007)	7.261	0.068 (0.004)	2.957
	% Reduction	51.1	9.7		9.3	
Sen = 0.85 Spe = 0.85	Baseline	0.047 (0.003)	0.183 (0.006)	3.894	0.074 (0.004)	1.574
	Reduced	0.023 (0.002)	0.166 (0.006)	7.217	0.068 (0.004)	2.957
	% Reduction	51.1	9.3		8.1	
Sen = 0.80 Spe = 0.80	Baseline	0.047 (0.003)	0.228 (0.006)	4.851	0.097 (0.005)	2.064
	Reduced	0.024 (0.002)	0.214 (0.006)	8.917	0.091 (0.005)	3.792
	% Reduction	51.1	6.1		6.2	
Latin America						
Sen = 1.00 Spe = 1.00	Baseline	0.033 (0.003)	0.033 (0.003)	1.000	0.004 (0.001)	0.125
	Reduced	0.016 (0.002)	0.016 (0.002)	1.000	0.002 (0.001)	0.125
	% Reduction	51.5	51.5		50	
Sen = 0.95 Spe = 0.95	Baseline	0.033 (0.003)	0.080 (0.004)	2.424	0.022 (0.002)	0.667
	Reduced	0.016 (0.002)	0.065 (0.004)	4.063	0.017 (0.002)	1.063
	% Reduction	51.5	18.7		22.7	
Sen = 0.95 Spe = 0.90	Baseline	0.033 (0.003)	0.128 (0.005)	3.879	0.046 (0.003)	1.394
	Reduced	0.016 (0.002)	0.114 (0.005)	7.125	0.040 (0.003)	2.500
	% Reduction	51.5	10.9		13.0	
Sen = 0.90 Spe = 0.90	Baseline	0.033 (0.003)	0.126 (0.005)	3.818	0.045 (0.003)	1.364
	Reduced	0.016 (0.002)	0.113 (0.005)	7.063	0.040 (0.003)	2.500
	% Reduction	51.5	10.3		11.1	
Sen = 0.90 Spe = 0.85	Baseline	0.033 (0.003)	0.175 (0.006)	5.303	0.070 (0.004)	2.121
	Reduced	0.016 (0.002)	0.162 (0.006)	10.125	0.065 (0.004)	4.063
	% Reduction	51.5	7.4		7.1	
Sen = 0.85 Spe = 0.85	Baseline	0.033 (0.003)	0.173 (0.006)	5.242	0.069 (0.004)	2.091
	Reduced	0.016 (0.002)	0.161 (0.005)	10.062	0.064 (0.004)	4.000
	% Reduction	51.5	6.9		7.2	
Sen = 0.80 Spe = 0.80	Baseline	0.033 (0.003)	0.219 (0.006)	6.636	0.093 (0.005)	2.818
	Reduced	0.016 (0.002)	0.209 (0.006)	13.064	0.089 (0.004)	5.563
	% Reduction	51.5	4.5		4.3	

Type-specific prevalence at baseline, in the United States: HPV16 = 0.047; in Latin America: HPV16 = 0.033

Reduced: HPV16 is reduced by 50 %

Abbreviations: *SD* standard deviation, *Sen* sensitivity, *Spe* specificity

**Table 4:** Designated and estimated prevalence of HPV18 by Linear Assay (LA)-only and sequential testing strategies in the United States and Latin America

Genotyping assay performance	Time	Designated composite prevalence (SD)	LA only		Sequential testing	
			Composite prevalence estimate (SD)	Estimate-to-true ratio	Composite prevalence estimate (SD)	Estimate-to-true ratio
United States						
Sen = 1.00 Spe = 1.00	Baseline	0.019 (0.002)	0.019 (0.002)	1.000	0.003 (0.001)	0.158
	Reduced	0.009 (0.002)	0.009 (0.002)	1.000	0.002 (0.001)	0.222
	% Reduction	52	52		33	
Sen = 0.95 Spe = 0.95	Baseline	0.019 (0.002)	0.067 (0.004)	3.526	0.020 (0.002)	1.053
	Reduced	0.009 (0.002)	0.058 (0.004)	6.444	0.017 (0.002)	1.889
	% Reduction	52	13.4		15	
Sen = 0.95 Spe = 0.90	Baseline	0.019 (0.002)	0.116 (0.005)	6.105	0.043 (0.003)	2.263
	Reduced	0.009 (0.002)	0.108 (0.005)	12.000	0.040 (0.003)	4.444
	% Reduction	52	6.9		7	
Sen = 0.90 Spe = 0.90	Baseline	0.019 (0.002)	0.115 (0.005)	6.052	0.042 (0.003)	2.210
	Reduced	0.009 (0.002)	0.107 (0.005)	11.889	0.039 (0.003)	4.333
	% Reduction	52	7.0		7.1	
Sen = 0.90 Spe = 0.85	Baseline	0.019 (0.002)	0.164 (0.006)	8.632	0.066 (0.004)	3.474
	Reduced	0.009 (0.002)	0.157 (0.006)	17.444	0.064 (0.004)	7.111
	% Reduction	52	4.3		3.0	
Sen = 0.85 Spe = 0.85	Baseline	0.019 (0.002)	0.163 (0.006)	8.579	0.066 (0.004)	3.474
	Reduced	0.009 (0.002)	0.156 (0.006)	17.33	0.063 (0.004)	7.000
	% Reduction	52	4.3		4.5	
Sen = 0.80 Spe = 0.80	Baseline	0.019 (0.002)	0.211 (0.006)	11.105	0.090 (0.004)	4.737
	Reduced	0.009 (0.002)	0.206 (0.006)	22.889	0.088 (0.005)	9.778
	% Reduction	52	2.3		2.2	
Latin America						
Sen = 1.00 Spe = 1.00	Baseline	0.012 (0.002)	0.012 (0.002)	1.000	0.003 (0.001)	0.250
	Reduced	0.006 (0.002)	0.006 (0.002)	1.000	0.001 (0.001)	0.167
	% Reduction	50	50		50	
Sen = 0.95 Spe = 0.95	Baseline	0.012 (0.002)	0.061 (0.004)	5.083	0.016 (0.002)	1.333
	Reduced	0.006 (0.002)	0.056 (0.004)	9.333	0.015 (0.002)	2.500
	% Reduction	50	8.2		6.3	
Sen = 0.95 Spe = 0.90	Baseline	0.012 (0.002)	0.110 (0.005)	9.167	0.039 (0.003)	3.250
	Reduced	0.006 (0.001)	0.105 (0.005)	17.500	0.037 (0.003)	6.167
	% Reduction	50	4.5		5.1	
Sen = 0.90 Spe = 0.90	Baseline	0.012 (0.002)	0.109 (0.005)	9.083	0.039 (0.003)	3.250
	Reduced	0.006 (0.001)	0.105 (0.005)	17.500	0.037 (0.003)	6.167
	% Reduction	50	3.7		5.1	
Sen = 0.90 Spe = 0.85	Baseline	0.012 (0.002)	0.159 (0.006)	13.250	0.063 (0.004)	5.250
	Reduced	0.006 (0.001)	0.154 (0.006)	25.667	0.062 (0.004)	10.333
	% Reduction	50	3.1		1.6	
Sen = 0.85 Spe = 0.85	Baseline	0.012 (0.002)	0.158 (0.006)	13.167	0.063 (0.004)	5.250
	Reduced	0.006 (0.001)	0.154 (0.005)	25.667	0.061 (0.004)	10.166
	% Reduction	50	3.1		3.1	
Sen = 0.80 Spe = 0.80	Baseline	0.012 (0.002)	0.207 (0.006)	17.250	0.087 (0.004)	7.250
	Reduced	0.006 (0.001)	0.204 (0.006)	34.000	0.086 (0.004)	14.333
	% Reduction	50	1.1		1.1	

Type-specific prevalence at baseline, in the US: HPV18 = 0.019; in Latin America: HPV18 = 0.012

Reduced: HPV18 is reduced by 50 %

Abbreviations: *SD* standard deviation, *Sen* sensitivity, *Spe* specificity

## Discussion

With >80 % of the cervical cancer-related deaths occurring in low-resource countries where women in most of these countries typically do not have access to effective screening or treatment before, successful introduction of using HPV-clinical test to screen cervical cancer is substantially beneficial in identifying women at risk of developing severe cervical cell abnormalities for cervical cancer prevention [3]. Availability of using HPV-clinical testing to screen cervical cancer in low-resource countries might allow public health officials to use the data to monitor HPV infection in ways not previously possible. Since HPV diagnostic testing strategies for epidemiological monitoring and cervical cancer screening have been considered separately, we have used a Monte Carlo simulation approach to investigate how well the apparent prevalence based on HC2-only and sequential testing strategies compared to LA-only measures the designated “true” prevalence. The results suggest that HPV clinical testing can be incorporated to monitor HPV prevalence or vaccine effectiveness and the apparent prevalence based on different testing strategies can be different.

Our simulation study is based on the assumption that analytical sensitivity of HC2 for detecting existence of the 14 high-risk HPV types is lower than LA-only but the analytical specificity is similar between the two. The HC2 clinical test is designed to detect high enough viral loads (i.e., clinically relevant viral loads). Analytical sensitivity of the LA is at the sub-picogram level of HPV DNA and analytical sensitivity of HC2 is at the picogram level [33–36]. Therefore, among infected subjects, the number of subjects who test LA positive is higher than the number of subjects who test HC2 positive. The magnitude of difference between analytical sensitivities of LA and HC2 depends on the distribution of the viral load of the infected subjects. When a large portion of the HPV infected subjects are with lower viral load (i.e., lower than the HC2 detection limit of 5,000 genomes), the analytical sensitivity of LA is much higher than HC2. When a smaller portion of the HPV infected subjects is with lower viral load, the analytical sensitivity of LA is closer to the analytical sensitivity of HC2. In our study, the association between HC2 and LA test results is based on samples from 8,403 women in the U.S. NHANES (2003 to 2010) studies for whom both LA and HC2 test results collected. It is equivalent to the scenario of analytical sensitivity of HC2 being approximately 50 % lower than LA, but specificity being similar. We further investigate the scenario, HC2 and LA results are more agreeable, which is based on the pap cohort Study [37]. Among 1,849 women, 83 % of LA test-positive subjects were tested positive by using HC2, and 84 % of LA test-negative subjects were also tested negative by using HC2. This is equivalent to analytical sensitivity of HC2 for detecting the 14 high-risk types is approximately 15 % lower than LA, but analytical specificity is similar. As expected, the conclusions are similar (simulation results not shown).

For demonstration purpose, the 50 % reduction of type 16 and 18 prevalence is chosen in this simulation study. The estimated vaccine effectiveness (reduction in prevalence) in the literature [9] is much lower than 50 % because of low coverage rate. Since the objective is to examine three testing strategies when infection prevalence is reduced, the 50 % reduction serves the purpose.

We consider type-specific infections to be correlated because the risk factors of getting infected by various virus types are similar and subjects with weaker immune system are more likely to get infected and stay infected. The correlations between HPV types in this simulation study were set to be 0.06 which was based on the average value of 91 pairwise correlations of the LA genotyping results of the 14 high-risk HPV types in the 2003–2006 National Health and Nutrition Examination Surveys (NHANES).

Discussion of using PCR genotyping assay to monitor HPV is given elsewhere [27]. Estimated-to-true prevalence ratios using the LA-only testing strategy are high. It is because we still get enough false-positives in a low-prevalence setting even when specificity is as high as 0.95, therefore, overestimate the “true” prevalence substantially. Taking a type-specific HPV infection as an example, this result can be easily seen from the following equation:

Apparent prevalence = sensitivity*true prevalence + (1-specificity) * (1- true prevalence).

The influence of the “true” prevalence level on the magnitude of overestimation can also be seen by comparing results from the US with those form the Latin America. The magnitude of over-estimation is more severe in Latin America. This is because the “true” type-specific infection prevalence is generally lower in Latin America.

The simulation results suggest that clinical-HC2 can be used as a triage before genotype to monitor HPV prevalence. Compared with LA-only testing strategy, that estimated-to-true prevalence ratios using the sequential testing strategy are closer to 1. In addition, the sequential testing strategy might be more compatible with cervical cancer screening programs. HC2 results for women aged ≥30 years can be used to screen for cervical cancer. In contrast, subjects with positive LA results are neither informed nor referred for colposcopy. Also, sequential testing strategy might reduce costs by greatly reducing the number of genotyping tests [38].

The simulation results also reveal that HC2-only can be used to monitor HPV prevalence of the 14 high-risk types, despite HC2 having lower analytical sensitivity than LA. The estimated-to-true prevalence ratios of the 14 high-risk HPV types from an HC2-only testing strategy are closer to 1 than the LA-only testing strategy. When the designated “true” underlying vaccine-associated high-risk types (HPV16 and HPV18) are reduced by 50 %, similar to using the LA-only testing strategy, prevalence estimates of the 14 high-risk types from HC2-only testing strategy also decline. Either using the LA-only or the HC2-only testing strategy underestimates the designated “true” reduction.

Other possible benefits exist to incorporating the HC2 clinical test for monitoring HPV infections. HC2 (particularly, careHPV, the developed countries version of HC2) procedures are less complex and less labor-intensive, therefore, less subject to human error. HC2 testing using the automation provided by a rapid capture system can achieve high-volume throughput [39]. Also, performance (sensitivities and specificities) of commercially manufactured HC2 has been reported to be highly reliable and reproducible in cervical cancer screening setting [22, 40]. High repeatability and consistent performance of HC2 testing allows public health professionals to compare HPV prevalence estimates of the 14 high-risk types across different geographic areas.

The simulations were conducted on the basis of an assumption that analytical sensitivity of LA-only testing is higher than sensitivity of HC2-only testing. When PCR procedures are performed incorrectly according to standard protocols, performance of LA might be worse than the performance of HC2, which was not considered in our study. Additionally, we did not consider the scenarios of cross-protection or type replacement attributable to vaccination in this simulation study because evidence of cross-protection and type replacement is inconclusive [41]. Conducting a cost-benefit analysis to compare LA-only testing strategy with HC2 as a triage before LA for monitoring HPV prevalence among specific age groups or within regions might be beneficial. Bias introduced because of study design (e.g., sampling strategy), confounders (e.g., demographic characteristics or sexual behaviors), or missing data are also beyond the scope of this paper. Future studies are needed to investigate the impact of those factors. Future study can also be extended to incorporate the newly approved Cobas HPV-clinical test and nine-valent HPV vaccine.

## Conclusion

Estimated-to-true HPV infection prevalence ratios using LA-only testing strategy are generally higher than using HC2-only or using HC2 as a triage before genotype by LA. Clinical HPV test can be incorporated to monitor HPV infection prevalence. Data from screening through HPV-clinical test may be utilized for epidemiological monitoring. Caution is needed since the intervention effectiveness can be underestimated and the apparent prevalence from different testing strategies can be different.

## Abbreviations

DNA: Deoxyribonucleic acid
HC2: Hybrid Capture 2
HPV: Human papillomavirus
LA: Linear Array
PCR: Polymerase chain reaction
